# Stress phenotypes in epilepsy: impact on cognitive functioning and quality of life

**DOI:** 10.3389/fpsyg.2023.1100101

**Published:** 2023-06-14

**Authors:** Judit Catalán-Aguilar, Esperanza González-Bono, Alejandro Lozano-García, Paula Tormos-Pons, Kevin G. Hampel, Vicente Villanueva, Irene Cano-López

**Affiliations:** ^1^Institut d’Investigació en Psicologia dels Recursos Humans, del Desenvolupament Organitzacional i de la Qualitat de Vida Laboral (Idocal)/Department of Psychobiology, Psychology Center, Universitat de València, Valencia, Spain; ^2^Refractory Epilepsy Unit, Neurology Service Member of ERN EPICARE, Hospital Universitario y Politécnico La Fe, Valencia, Spain; ^3^Faculty of Health Sciences, Valencian International University, Valencia, Spain

**Keywords:** epilepsy, stress, resilience, memory, executive function, naming, cognition, quality of life

## Abstract

**Introduction:**

Drug-resistant epilepsy has been proposed as a chronic stress model. Stress can be measured in terms of chronicity (epilepsy duration) and intensity (comorbidities), with depression and anxiety among the most important comorbidities in epilepsy due to its prevalence and its relationship with cognitive functioning and quality of life. This study aims to establish phenotypes according to how patients face a stressful condition (epilepsy) and examine differences in cognition and quality of life depending on these phenotypes. We hypothesize that there will be an interrelationship between epilepsy duration and negative affectivity, and these variables will influence cognition and quality of life.

**Methods:**

170 patients (82 men and 88 women) underwent a neuropsychological evaluation in which trait anxiety, depression, attention and executive function, verbal and visual memory, language, emotional recognition, and quality of life were assessed. Hierarchical clustering was performed using z-scores for three variables: trait anxiety; depression; and epilepsy duration.

**Results:**

Three clusters were found: vulnerable (high negative affectivity and short duration); resilient (moderate negative affectivity and long duration); and low-impact group (low negative affectivity and short duration). Results show that the vulnerable group had poorer cognitive functioning and quality of life than the other groups. Specifically, the vulnerable group had poorer scores than the low-impact group on verbal memory, visual confrontation naming, and quality of life (except seizure worry). Furthermore, resilient patients had better scores than the low-impact group on cognitive flexibility variables, but lower scores on some quality-of-life subscales (i.e., overall quality of life, emotional well-being, and energy). Finally, the vulnerable group had poorer scores than the resilient group in executive functioning, naming, and quality of life.

**Discussion:**

These results suggest that dealing with stress in patients with epilepsy is related to cognitive performance and quality of life. These findings underline the relevance of considering comorbidities in epilepsy and may be useful for detecting vulnerable or resilient profiles as risk or protective factors for cognitive and quality of life decline.

## 1. Introduction

Epilepsy is a disease that affects almost 1% of the general population and is characterized by the recurrence of seizures and its global impact on the individual (Fiest et al., 2017). Seizures have been considered an acute stressor, within the framework of epilepsy as a potentially chronic stress state (Cano-López and Gonzalez-Bono, 2019). Stress has been defined as “a process in which an individual perceives that demands exceed the organism’s regulatory capacity to adapt to a psychological or physiological challenge or stressor” (Cano-López and Gonzalez-Bono, 2019). The impact of the stress process on the individual can be measured in terms of chronicity or intensity.

Regarding chronicity, seizures are stressful events that lead to the hypersecretion of cortisol in patients with epilepsy (Cano-López and Gonzalez-Bono, 2019; Brandner et al., 2022). Therefore, prolonged exposure to seizures can lead to a situation of chronic stress. In this regard, the number of years suffering from the disease (i.e., epilepsy duration) is a measure that reflects the chronicity of the stressor (i.e., epilepsy) and it may have implications on different areas of the individual. Longer periods of epilepsy duration have been related to poorer verbal memory functioning in patients with temporal lobe epilepsy (TLE) (Kent et al., 2006), which is the main cognitive concern in this population (Helmstaedter and Kockelmann, 2006; Thompson et al., 2016). Similarly, several studies have found that longer epilepsy duration is related to poorer executive function (Kim et al., 2007; Black et al., 2010; Zamarian et al., 2011). Bonilha et al. (2006) suggested that longer epilepsy duration may imply greater gray matter loss, and this may lead to further cognitive impairment. Nevertheless, the relationship between epilepsy duration and health-related quality of life (QOL) has been found to be inconsistent (Taylor et al., 2011) although several studies have found that longer epilepsy duration is related to poorer QOL (Piperidou et al., 2008; Edefonti et al., 2011; Pauli et al., 2012).

Stress intensity is a more complex factor since it may include seizure frequency and the comorbidities of epilepsy—the presence of which is being increasingly recognized by the International League Against Epilepsy (ILAE) (Scheffer et al., 2017). The difficulty of reliable recording of the seizure frequency should be emphasized, since this information is usually collected by patients and relatives, but patients are not always aware of when they have seizures, or sometimes even forget to write them down (Blachut et al., 2017). For this reason, although this variable offers an approximate value, it cannot be considered a precise variable, especially when the number of seizures is very high. Negative affectivity is considered one of the most frequent comorbidities in patients with epilepsy (Fiest et al., 2013; Kwon and Park, 2014; Park, 2016; Pham et al., 2017) and provides a measure of the interpretation that the individual makes of the stressful event. It has been conceptualized as an exacerbated emotional response to unpredictable seizures (including depression and anxiety), as well as to activity restriction, which in turn leads to low self-esteem, stigma, and social rejection (Kotwas et al., 2017). It has been suggested that negative affectivity would be a more integrative concept than seizure frequency, overlapping with the seizure frequency in some aspects and providing further information even on stressful life events (i.e., factors related and not related to epilepsy) (Kotwas et al., 2017). Specifically, negative affectivity is directly related to clinical variables such as seizure frequency or adverse effects derived from anti-seizure medications (ASMs) (Thapar et al., 2009; Kanner et al., 2012; Dehn et al., 2017), as well as with other stress variables such as social stigma (Thapar et al., 2009), so it may have an even more important contribution to impaired QOL in this population (Kotwas et al., 2017). Thus, it has been found that stigma is strongly correlated to more depressive symptoms and poorer QOL in people with epilepsy (Tombini et al., 2019). Furthermore, negative affectivity is also linked to other psychosocial processes, being alexithymia (i.e., a subclinical phenomenon involving a lack of emotional awareness) an important concept due to its high prevalence epilepsy (Tojek et al., 2000; Bewley et al., 2005; Myers et al., 2013). Specifically, people with epilepsy with higher levels of anxiety or depression are more likely to experience alexithymia (Tombini et al., 2020a; Choi et al., 2021). In this regard, Tombini et al. (2020a) suggested that alexithymia could be considered a symptom of depression, or even alexithymia could be considered a risk factor for depression, given the scarcity of coping strategies for dealing with hard-to-identify emotions in these patients. Finally, negative affectivity has also been related to poor cognitive functioning and QOL in patients with epilepsy (Tracy et al., 2007; Kanner et al., 2010; Cano-López et al., 2018; Cano-López, 2019) suggesting that this variable holistically affects individuals with epilepsy. Therefore, it is not surprising that patients with epilepsy show lower life satisfaction than people without epilepsy, which could be explained, at least in part, by negative affectivity (Kang, 2023).

Negative affectivity can be considered a factor involved in stress intensity and as a key variable related to coping with the disease. Risk or protection profiles for coping with the disease have been developed considering emotional and other epilepsy-related variables. An example of this is the model proposed by Ring et al. (2016) using qualitative research methods which differentiate vulnerable and resilient individuals and explain how different socioemotional and clinical factors influence QOL in patients with epilepsy. Furthermore, Tedrus et al. (2020) suggest that resilience, which can be considered as the ability to cope with a stressful situation satisfactorily (Zapater-Fajarí et al., 2021), is a protective factor for mood disorders in patients with epilepsy. To date, however, this approach has considered the chronicity of the alterations as an extraneous variable to be controlled and not as a measure of the chronicity of the stressor.

Studies focused on the relationship between epilepsy duration and negative affectivity offer heterogeneous results. A recent meta-analysis carried out by Yang et al. (2020) showed that patients with a shorter epilepsy duration had a lower risk of depression. The meta-analysis by Scott et al. (2020) also found that shorter epilepsy duration was associated with a lower prevalence of depressive and anxiety disorders in young people with epilepsy. Brandt (2016), however, found that younger patients with a shorter epilepsy duration were more likely to have anxiety disorders, underlying that an adaptation period is required to develop coping strategies for the stressful situation. These results suggest that there is an interrelationship between epilepsy duration and negative affectivity, and these variables may influence cognition and QOL. However, as far as we know, no studies have summarized stress chronicity and intensity variables in different profiles, nor explored their association with cognition and QOL in the same sample of patients with epilepsy. For this reason, the present study aims to detect phenotypes based on indicators of stress chronicity and intensity (i.e., epilepsy duration, trait anxiety, and depression) in patients with drug-resistant epilepsy, as well as examine differences in cognition and QOL depending on these phenotypes. Although the longer duration of epilepsy and greater negative affectivity may be separately related to poorer cognitive functioning and poorer QOL, we hypothesize that there may be a profile of patients who have adapted adequately to the disease, despite having suffered from it for a long time, presenting moderate levels of negative affectivity. We hypothesize that this profile will be more adaptive at a cognitive and QOL level than the profile of patients with greater negative affectivity.

## 2. Materials and methods

### 2.1. Patients

This is a cross-sectional study in which participants were recruited from the Refractory Epilepsy Unit of the Hospital Universitario y Politécnico La Fe between April 2015 and November 2022. The inclusion criteria were: (a) patients with a diagnosis of drug-resistant epilepsy, thus ensuring that the disease was chronic; (b) candidates for epilepsy surgery to ensure relative homogeneity of the sample (i.e., suspected focal onset seizures); (c) a chronological age of at least 18, to ensure cognitive development; (d) and a neuropsychological assessment performed before surgery. Excluded were patients who: (a) were older than 65, to avoid the possible presence of sensory deficits and fatigue effects; (b) in whom the assessment could not be carried out with a minimum of reliability due to their high level of cognitive impairment; and (c) had a history of severe psychiatric conditions, since it could affect the results obtained in the neuropsychological evaluation.

### 2.2. Procedure

The study was conducted following the Declaration of Helsinki and approved by the ethics committee of the hospital. All participants provided informed consent. Our reporting followed STROBE guidelines (Von Elm et al., 2007).

Demographic characteristics (i.e., age, sex, handedness, educational level, academic/employment insertion, and household members), as well as clinical data (i.e., epilepsy type, TLE (yes/no), side of seizure focus, age at epilepsy onset, epilepsy duration, magnetic resonance imaging (MRI) findings, hippocampal sclerosis (HS) (yes/no), number of ASMs, and seizures per month and seizure type) were registered. In this regard and given the objectivity, robustness, and relevance of the variable in this study, epilepsy duration was collected during the interview before the neuropsychological assessment, in which the information provided by the patients was contrasted with the relatives. This variable considered the years elapsed from the time the diagnosis was made until the neuropsychological assessment was performed.

Presurgical assessment included the diagnosis of the epilepsy type and the lateralization of the epileptogenic area based on a comprehensive assessment made by members of a multidisciplinary team. This evaluation included seizure history and semiology, neurologic assessment, video-electroencephalography monitoring, 3-Tesla MRI, psychiatric assessment, and neuropsychological evaluation. Fluorodeoxyglucose (FDG)- positron emission tomography (PET), single-photon emission computed tomography (SPECT), and intracranial EEG recording were performed selectively. A comprehensive neuropsychological evaluation was carried out for all patients. From this assessment, anxiety, depression, attention, executive functioning, memory, language, and QOL tests were selected for the present study. Furthermore, to ensure that the differences in negative affectivity values in each group were not due to difficulties in emotion recognition, an instrument that assessed the ability to recognize emotional states was administered.

### 2.3. Neuropsychological assessment

The neuropsychological assessment was designed following the recommendations of the E-PILEPSY consortium (Vogt et al., 2017).

#### 2.3.1. Anxiety

This was assessed using the State–Trait Anxiety Inventory (STAI) (Spielberger, 1989). The trait anxiety scale (STAI-T) evaluates relatively stable aspects of anxiety and is composed of 20 items rated on a four-point scale ranging from 0 (“hardly never”) to 3 (“almost always”), with higher scores indicating higher anxiety. Cronbach’s alpha of the Spanish adaptation of this inventory is 0.94 (Guillén-Riquelme and Buela-Casal, 2011). This instrument was selected since, despite being a test developed for the general population, it is a valid and reliable instrument with acceptable sensitivity and specificity in patients with epilepsy, with a high negative predictive value and a low positive predictive value (Wiglusz et al., 2019; Zingano et al., 2019). It is a strong predictor for QOL in this population (Johnson et al., 2004; Cano-López et al., 2018). Moreover, it is one of the most widely used measures of anxiety in clinical research (Spielberger et al., 1970; Spielberger, 1983; Kennedy et al., 2001; Wiglusz et al., 2019), thus enabling us to have a large literature with which to compare our results (Cano-López et al., 2018; Lima et al., 2021; Cano-López et al., 2023).

#### 2.3.2. Depression

The Beck Depression Inventory-II (BDI-II) (Beck et al., 1996) was used to assess depression with 21 items rated on a four-point scale, with higher scores indicating higher depression levels. Cronbach’s alpha is 0.89 (Sanz and García-Vera, 2009). This instrument was selected since the E-PILEPSY consortium (Vogt et al., 2017) reported that the BDI was the wide instrument used by clinicians (60%) to assess mood in patients with drug-resistant epilepsy. Furthermore, it has been shown that BDI-II is a more robust measure than other instruments in patients with epilepsy (de Oliveira et al., 2014).

#### 2.3.3. Attention and executive functioning

The Trail Making Test (TMT) (Reitan and Wolfson, 1985) was used to measure attention and executive functions (working memory, attention, planning, and set-shifting) that require motor skills and visual–spatial processing. In part A (TMT-A), participants were requested to draw a line to connect 25 circles with successive numbers and in the correct order, whereas in part B (TMT-B) participants must alternate between letters, joining them in the specific order. In this test, longer task completion times indicate poorer performance.

The Wisconsin Card Sorting Test (WCST) (Heaton et al., 1993) was used to evaluate cognitive flexibility, abstract conceptualization, and responsiveness to feedback. Higher scores indicated poorer performance in the following indices: number of trials; number and percentage of errors; number and percentage of perseverative responses; number and percentage of non-perseverative errors; trials to complete the first category; and failure to maintain a set. Additionally, higher scores showed better performance in the following indices: correct responses; number and percentage of conceptual level responses; categories completed; and learning to learn.

#### 2.3.4. Memory

The Spanish Complutense Verbal Learning Test (TAVEC) (Benedet and Alejandre, 1998) was used to assess episodic verbal memory. This test is a Spanish version of the California Verbal Learning Test (CVLT) (Delis et al., 1987). It consists of three shopping lists: a learning list (list A); an interference list (list B); and a recognition list (list C). The following indices were computed: immediate verbal memory; short-term verbal memory; short-term verbal memory with semantic cues; long-term verbal memory; long-term verbal memory with semantic cues; recognition; and discriminability. In all cases, higher scores indicated better performance.

The Rey-Osterrieth Complex Figure test (ROCF) (Rey, 1941; Osterrieth, 1944) was used to assess immediate visual memory. It consists of the presentation of a two-dimensional figure that must be copied by the patient, without rotating the model sheet. After 3 min, participants were asked to recall the figure and draw it again without the presence of the model, assessing the immediate visual memory. The scores were computed as the sum of the drawn elements considering the degree of accuracy, deformation, and location. According to this correction system, each of the 18 elements of the figure received a score of 0, 0.5, 1, or 2 points. The maximum possible score was 36 points.

#### 2.3.5. Language functions

The Boston Naming Test (Kaplan et al., 2001) was used to assess visual confrontation naming. Semantic and phonemic cues were provided in the case of no response or incorrect response. The total score was computed as the number of cards correctly named without phonemic cues and with 60 being the maximum score.

The FAS (Spreen and Benton, 1977) was used to assess phonemic fluency. In this task, participants were requested to say as many words as they can that start with the letters F, A, and S in 1 min. The total score was computed as the sum of all admissible words for the three letters.

The Animal Naming Test (ANT) (Rosen, 1980) was used to evaluate semantic fluency. In this test, participants were required to name as many animals as possible in 1 min. The total score was computed as the sum of admissible words for this semantic category.

#### 2.3.6. Emotional recognition

The Reading the Mind in the Eyes Test (RMET) (Baron-Cohen et al., 2001) was used in its Spanish version (Fernández-Abascal et al., 2013). This test measures the ability to recognize other people’s emotions. It consists of 36 photographs of people’s gazes, each with four response options. Participants are asked to choose which adjective best describes that look. The test score ranges from 0 to 36, the higher the score, the better the performance. Cronbach’s alpha of the Spanish adaptation is 0.63.

#### 2.3.7. QOL

Quality-of-Life in Epilepsy Inventory (QOLIE-31) (Cramer et al., 1998), in its Spanish version (Torres et al., 1999), was used to assess QOL and includes 31 items distributed in seven scales: seizure worry; overall QOL; emotional wellbeing; energy; cognitive self-rating; medication effects; and social functioning. Scores for each subscale ranged from 0 to 100, with higher scores indicating better QOL (including seizure worry and medication effects, which were scored on an inverse scale). A QOL composite score was computed using a weighted average of subscales. Cronbach’s alpha of the Spanish adaptation of this inventory ranges from 0.55 to 0.92 (Torres et al., 1999).

### 2.4. Statistical analysis

Outliers were defined as values ±2.5 SD. We detected three outliers for BDI-II and one for epilepsy duration, and they were winsorized by replacing their values with values equal to the mean ± 2.5 SD to control for the possible effect of extreme values in further analyses (Dixon and Tukey, 1968). A cluster analysis was used to identify groups of participants based on their average z*-*scores across three variables: anxiety; depression; and epilepsy duration. We used Ward’s hierarchical clustering method with squared Euclidean distance as the index of the length between patient profiles. This method is one of the most widely used (Govender and Sivakumar, 2020) probably due to its effectiveness at the time of classification (Tufféry, 2011). Ward’s method aims to achieve the minimization of intragroup variance and maximizes homogeneity within groups. This analysis provides groups or clusters not defined *a priori*. Hierarchical clusters provide an output called dendrogram—in which the progressive formation of clusters is shown from n subjects to a single grouping. The R package NbClust was used to check the optimal number of clusters. This package is particularly useful as it provides 23 indices and indicates how many indices support a given number of clusters after varying all combinations of several clusters, distance measures, and clustering methods (Charrad et al., 2013). Dunn’s index, which measures the ratio of the minimum inter-cluster distance to the maximum inter-cluster distance, was also calculated. The value of this index ranges from 0 to infinity with higher scores indicating better clustering. Finally, the analyses were replicated with a k-means cluster to observe agreement with the results of the hierarchical cluster. These analyses were made using Rstudio (version 4.0.0).^1^

Once the clusters were established, univariate ANOVAs were performed for between-group comparisons based on the cluster and clinical and demographical variables (i.e., age, educational level, age at epilepsy onset, epilepsy duration, number of ASMs, and seizures per month). Moreover, the chi-square test was used to study the differences between frequencies in descriptive variables depending on the cluster [i.e., sex, handedness, academic/employment insertion, household members, epilepsy type, TLE (yes/no), side of seizure focus, MRI findings, HS (yes/no) and seizure type]. When significant differences were detected, these demographical and clinical variables were included as covariates in further analyses. Univariate ANOVAs were performed using the z-scores of each variable to determine whether these clusters differed for attention and executive functioning, memory, language, QOL, and emotional recognition. Bonferroni tests were then performed as *post hoc* analyses. ANOVAs were performed using SPSS 25.0 and two-tailed tests with *p* set at 0.05 were considered significant. Partial eta squared effect sizes were reported, but this indicator may be difficult to interpret when comparing between studies (Lakens, 2013). Consequently, and considering the recommendation of Preacher and Kelley (2011) of the use of multiple effect size measures, Cohen’s *f* statistic was calculated as a local effect size measure using G*Power 3.1.9.7 software, with values near 0.10, 0.25, and 0.40 representing small, medium, and large effect sizes, respectively (Cohen, 1969).

## 3. Results

### 3.1. Characteristics of the total sample

The sample was composed of 170 patients (82 men and 88 women; mean age = 38.21, *SD* = 11.20). The mean age at epilepsy onset was 16.60 (*SD* = 11.86) and the mean of seizures per month was 20.49 (*SD* = 44.47). Characteristics of the sample are shown in Table 1.

**Table 1:** Characteristics of the total sample and clusters [mean ± SD or n (%)].

	Vulnerable phenotype (*n* = 45)	Resilient phenotype (*n* = 59)	Low-impact phenotype (*n* = 66)	Total (*n* = 170)	*p*
Age (years)	36.04 ± 9.88	47.98 ± 8.73	33.58 ± 9.56	38.21 ± 11.20	0.0001
Sex					0.73
Male	24 (53.3%)	22 (46.8%)	36 (46.2%)	82 (48.2%)	
Female	21 (46.7%)	25 (53.2%)	42 (53.8%)	88 (51.8%)	
Handedness					0.48
Right	37 (82.2%)	44 (93.6%)	70 (89.7%)	151 (88.8%)	
Left	5 (11.1%)	2 (4.3%)	4 (5.1%)	11 (6.5%)	
Mixed	3 (6.7%)	1 (2.1%)	4 (5.1%)	8 (4.7%)	
Educational level					0.01
Primary	7 (15.6%)	5 (10.6%)	6 (7.7%)	18 (10.6%)	
Secondary	20 (44.4%)	30 (63.8%)	27 (34.6%)	77 (45.3%)	
Lower university	10 (22.2%)	7 (14.9%)	26 (33.3%)	43 (25.3%)	
University	8 (17.8%)	5 (10.6%)	19 (24.4%)	32 (18.8%)	
Academic/employment insertion					0.06
Yes	19 (42.2%)	21 (44.7%)	48 (61.5%)	88 (51.8%)	
No	26 (57.8%)	26 (55.3%)	30 (38.5%)	82 (48.2%)	
Household members					0.11
Family	16 (35.6%)	9 (19.15%)	34 (43.6%)	59 (34.7%)	
Partner	23 (51.1%)	32 (68.1%)	38 (48.7%)	93 (54.7%)	
Flatmate	2 (4.4%)	0 (0%)	1 (1.3%)	3 (1.8%)	
Living alone	4 (8.9%)	6 (12.8%)	5 (6.4%)	15 (8.8%)	
Epilepsy type					0.51
FLE^a^	6 (13.3%)	11 (23.4%)	16 (20.5%)	33 (19.4%)	
TLE^b^	34 (75.6%)	34 (66.1%)	51 (77.3%)	119 (70%)	
PLE^c^	3 (6.7%)	1 (2.1%)	8 (10.3%)	12 (7.1%)	
OLE^d^	1 (2.2%)	0 (0%)	1 (1.3%)	2 (1.2%)	
ILE^e^	0 (0%)	1 (2.1%)	0 (0%)	1 (0.6%)	
Multifocal	1 (2.2%)	0 (0%)	2 (2.6%)	3 (1.8%)	
TLE^b^ (yes/no)					0.46
Yes	34 (75.6%)	34 (72.3%)	51 (65.4%)	119 (70%)	
No	11 (24.4%)	13 (27.7%)	27 (34.6%)	51 (30%)	
Side of seizure focus				0.86	
Left	23 (51.1%)	23 (48.9%)	34 (43.6%)	80 (47.1%)	
Right	20 (44.4%)	22 (46.8%)	38 (48.7%)	80 (47.1%)	
Bilateral	2 (4.4%)	2 (4.3%)	6 (7.7%)	10 (5.9%)	
Age at epilepsy onset (years)	21.62 ± 11.36	7.23 ± 6.47	19.36 ± 11.56	16.60 ± 11.86	0.0001
Epilepsy duration (years)	14.42 ± 9.07	40.75 ± 8.77	14.22 ± 8.72	21.61 ± 14.76	0.0001
MRI^f^ findings					0.009
HS^g^	10 (22.2%)	23 (48.9%)	16 (20.5%)	49 (28.8%)	
FCD^h^	5 (11.1%)	13 (27.7%)	18 (23.1%)	36 (21.2%)	
Tumor	10 (22.2%)	2 (4.3%)	13 (16.7%)	25 (14.7%)	
Heterotopia	2 (4.4%)	0 (0%)	3 (3.8%)	5 (2.9%)	
Cavernona	5 (11.1%)	2 (4.3%)	8 (10.3%)	15 (8.8%)	
Non-specific pathology	13 (28.9%)	7 (14.9%)	20 (25.6%)	40 (23.5%)	
HS^g^ (yes/no)					0.002
Yes	10 (22.2%)	23 (48.9%)	16 (20.5%)	49 (28.8%)	
No	35 (77.8%)	24 (51.1%)	62 (79.5%)	121 (71.2%)	
Number of ASMs^i^	2.76 ± 0.88	3.21 ± 0.93	2.71 ± 0.81	2.86 ± 0.89	0.006
Seizures per month	26.05 ± 50.77	22.95 ± 42.62	17.15 ± 42.09	20.49 ± 44.48	0.583
Seizure type					0.498
FAS^j^	1 (2.2%)	0 (0%)	6 (7.7%)	7 (4.1%)	
FIAS^k^	15 (33.3%)	22 (46.8%)	24 (30.8%)	61 (35.9%)	
FBTCS^l^	1 (2.2%)	0 (0%)	2 (2.6%)	3 (1.8%)	
FAS^j^ + FIAS^k^	9 (20%)	10 (21.3%)	13 (16.7%)	32 (18.8%)	
FAS^j^ + FBTCS^l^	2 (4.4%)	0 (0%)	3 (3.8%)	5 (2.8%)	
FIAS^k^ + FBTCS^l^	13 (28.9%)	10 (21.3%)	23 (29.5%)	46 (27.1%)	
FAS^j^ + FIAS^k^ + FBTCS^l^	4 (8.9%)	5 (10.6%)	7 (9%)	16 (9.4%)	
RMET^m^	18.81 ± 5.88	18.84 ± 5.42	20.53 ± 4.70	19.62 ± 5.27	0.318

^a^FLE, frontal lobe epilepsy; ^b^TLE, temporal lobe epilepsy; ^c^PLE, parietal lobe epilepsy; ^d^OLE, occipital lobe epilepsy; ^e^ILE, insular lobe epilepsy; ^f^MRI, magnetic resonance imaging; ^g^HS, hippocampal sclerosis; ^h^FCD, focal cortical dysplasia; ^i^ASM, antiseizure medication; ^j^FAS, focal aware seizure; ^k^FIAS, focal impaired awareness seizure; ^l^FBTCS, focal to bilateral tonic–clonic seizures; ^m^RMET, Reading the Mind in the Eyes Test.

### 3.2. Stress phenotypes

**Figure 1:**
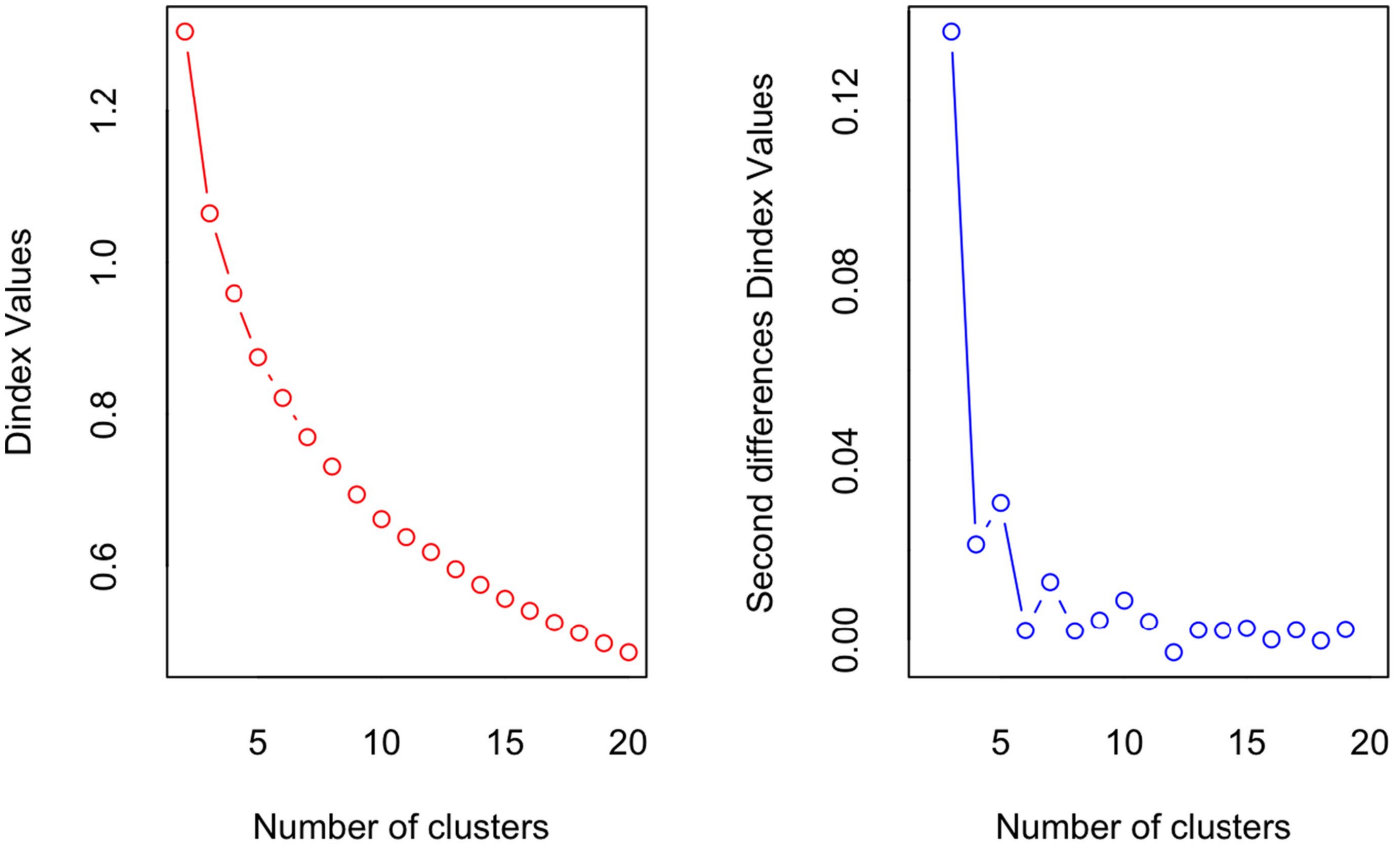
D-index for the determination of the number of clusters. The sharp change in the slope of the D-index second differences plot indicates a significant increase in the value of the measure.

Fourteen of the 23 indices from NbClust R package indicated that a three-cluster solution was an optimal number of clusters for portioning the data. The Dunn index for a three-cluster solution was 1.07. When the K-means cluster was performed with a three-cluster solution, the same grouping of patients was obtained for each of the three clusters. Figure 1 shows the Dunn index plot.

Cluster 1 (i.e., vulnerable phenotype) comprised 26.47% of patients and was characterized by high anxiety and depression, but short epilepsy duration (mean = 14.42 years, *SD* = 9.07). Cluster 2 (i.e., resilient phenotype) included 27.65% of the patients and was characterized by moderate levels of anxiety and depression, and long epilepsy duration (mean = 40.75 years, *SD* = 8.77). Cluster 3 (i.e., low-impact phenotype) comprised 45.88% of patients and was considered as a relative control group as patients in this group had low levels of anxiety, depression, and short durations (mean = 14.22 years, *SD* = 8.72). Mean z-scores and SD for each group are shown in Table 2. As expected, significant differences were found in epilepsy duration, anxiety, and depression depending on the cluster (for all, *p* < 0.0001).

**Table 2:** Mean and SD of each cluster based on z scores.

	Vulnerable phenotype (*n* = 45)	Resilient phenotype (*n* = 47)	Low-impact phenotype (*n* = 78)	Differences between groups
BDI-II	1.06 ± 0.83	−0.04 ± 0.75	−0.60 ± 0.53	*p* < 0.0001
STAI-R	1.09 ± 0.52	0.07 ± 0.77	−0.65 ± 0.74	*p* < 0.0001
Epilepsy duration	−0.47 ± 0.62	1.32 ± 0.59	−0.49 ± 0.59	*p* < 0.0001

### 3.3. Patient characteristics depending on stress phenotypes

Patient characteristics depending on stress phenotypes are shown in Table 1. Significant differences depending on stress phenotypes were found for age [*F*_(2, 169)_ = 35.84, *p* < 0.0001] with the resilient group being significantly older than the vulnerable and low-impact groups (*p* < 0.0001). Significant differences were also found in age for epilepsy onset [*F*_(2, 169)_ = 27.33, *p* < 0.0001], resilient patients having an earlier onset than patients from vulnerable and low-impact phenotypes (*p* < 0.0001); and for educational level [*F*_(2, 169)_ = 4.72, *p* = 0.01] with resilient patients showing lower educational levels than the low-impact group (*p* = 0.011). Furthermore, significant differences were found in the number of ASMs [*F*_(2, 168)_ = 5.34, *p* = 0.006] with resilient patients taking more ASMs than the other phenotypes (for both, *p* ≤ 0.036). Finally, significant differences were found in MRI findings with resilient patients having HS more frequently than the other groups (*p* = 0.002). Age, educational level, number of ASMs, and HS (yes/no) were included as covariates in further analyses to control the possible influence of these variables. Age of onset of epilepsy was not included as a covariate since it was directly related to epilepsy duration (i.e., the variable of interest in this study). No other significant differences were found in demographical or clinical variables (e.g., seizure frequency).

It should be noted that no differences were found depending on stress phenotypes in the RMET total score. Furthermore, all groups achieved a hit rate of more than 50%, so patients did not seem to have significant difficulties in perceiving and identifying emotions.

### 3.4. Differences in attention, executive functioning, memory, and language depending on stress phenotypes

**Figure 2:**
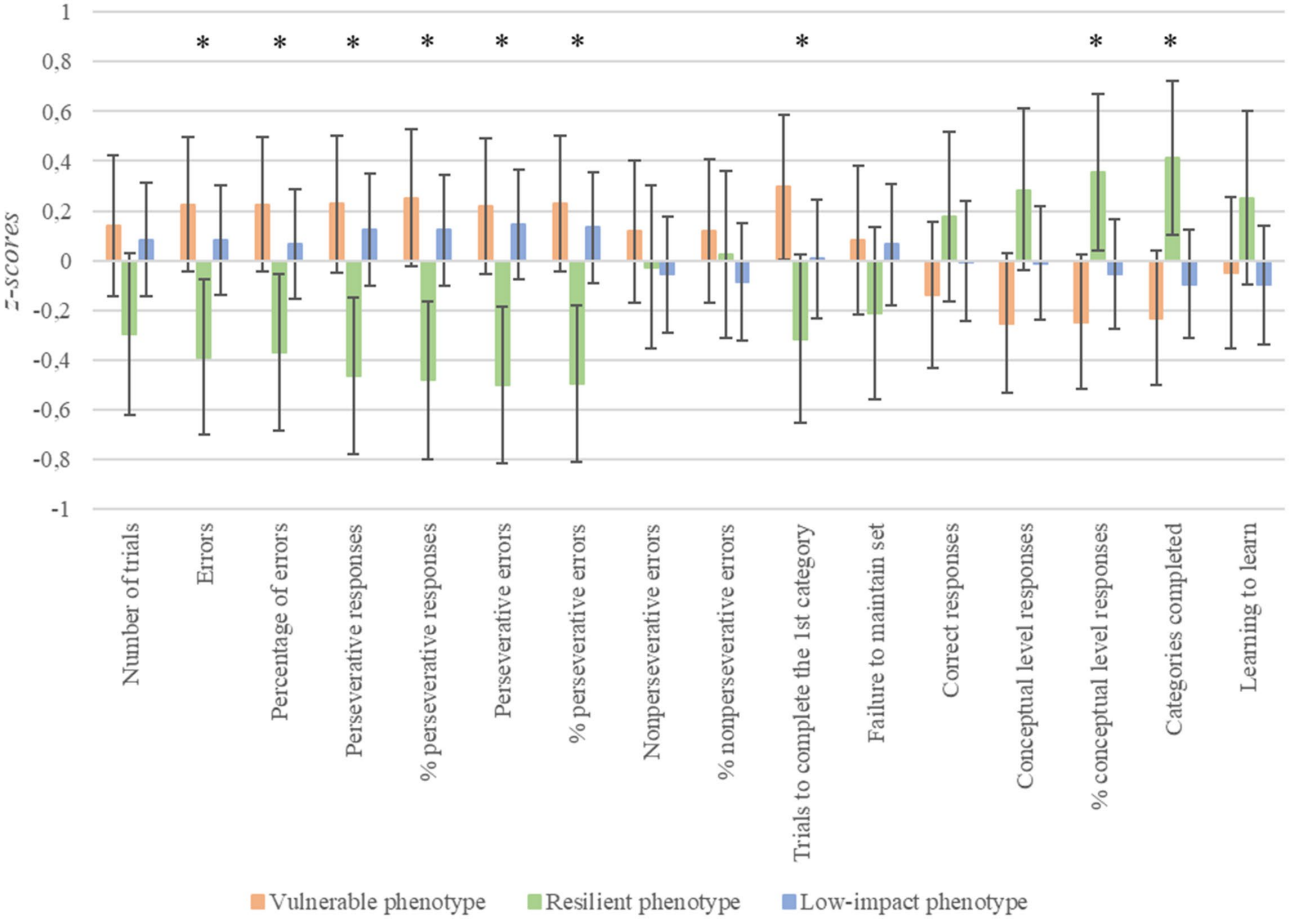
Differences in cognitive flexibility depending on stress phenotypes. ^*^p < 0.05; error bars represent 95% confidence intervals. Higher scores indicate poorer performance in most variables (from number of trials to failure to maintain set). However, higher scores indicate better performance in the case of correct responses, conceptual level responses, categories completed, and learning to learn.

Regarding executive functioning (Figure 2), significant differences were also found in the following WCST variables: errors [*F*_(2, 168)_ = 4.09, *p* = 0.019, *n*^2^*_p_* = 0.05, Cohen’s *f* = 0.24]; percentage of errors [*F*_(2, 168)_ = 3.79, *p* = 0.025, *n*^2^*_p_* = 0.05, Cohen’s *f* = 0.23]; perseverative responses [*F*_(2, 168)_ = 5.29, *p* = 0.006, *n*^2^*_p_* = 0.06, Cohen’s *f* = 0.29]; percentage of perseverative responses [*F*_(2, 168)_ = 5.83, *p* = 0.004, *n*^2^*_p_* = 0.07, Cohen’s *f* = 0.30]; perseverative errors [*F*_(2, 168)_ = 6.08, *p* = 0.003, *n*^2^*_p_* = 0.07, Cohen’s *f* = 0.31]; percentage of perseverative errors [*F*_(2, 168)_ = 5.99, *p* = 0.003, *n*^2^*_p_* = 0.07, Cohen’s *f* = 0.31]; percentage of conceptual level responses [*F*_(2, 168)_ = 3.81, *p* = 0.024, *n*^2^*_p_* = 0.045, Cohen’s *f* = 0.23]; completed categories [*F*_(2, 168)_ = 4.61, *p* = 0.011, *n*^2^*_p_* = 0.054, Cohen’s *f* = 0.24]; and trials to completed the first category [*F*_(2, 168)_ = 3.54, *p* = 0.031, *n*^2^*_p_* = 0.04, Cohen’s *f* = 0.23]. In these variables, vulnerable patients had significantly poorer performance than resilient patients (for all, *p* < 0.03). It should be noted that a tendency was also observed on conceptual level responses [*F*_(2, 168)_ = 2.88, *p* = 0.059, *n*^2^*_p_* = 0.03], specifically vulnerable patients performing worse than resilient patients (*p* = 0.056). Furthermore, the low-impact group performed significantly worse than the resilient group on perseverative responses, percentage of perseverative responses, perseverative errors, percentage of perseverative errors, and completed categories (for all, *p* < 0.05).

**Figure 3:**
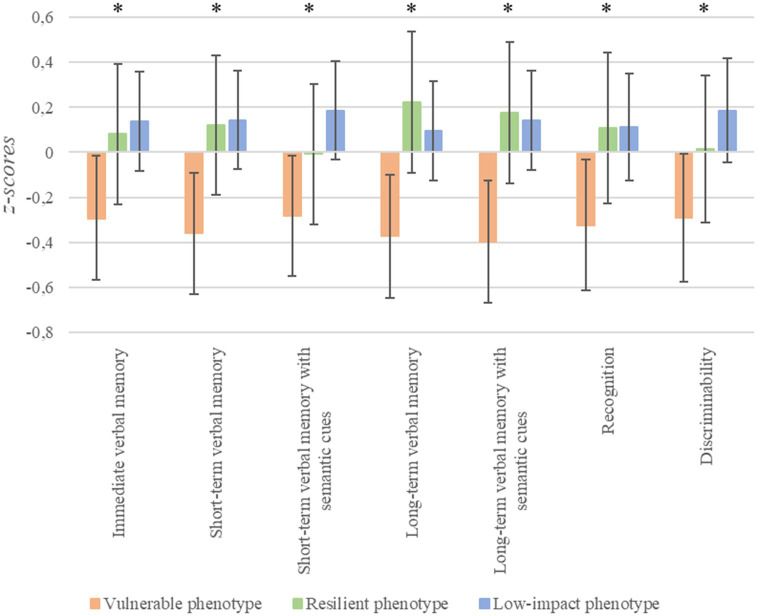
Differences in memory depending on stress phenotypes. ^*^p < 0.05; error bars represent 95% confidence intervals.

With respect memory (Figure 3), significant differences were found in the following TAVEC variables: immediate verbal memory [*F*_(2, 168)_ = 3.41, *p* = 0.036, *n*^2^*_p_* = 0.04, Cohen’s *f* = 0.18]; short-term verbal memory [*F*_(2, 168)_ = 4.86, *p* = 0.009, *n*^2^*_p_* = 0.06, Cohen’s *f* = 0.22]; short-term verbal memory with semantic cues [*F*_(2, 168)_ = 3.69, *p* = 0.027, *n*^2^*_p_* = 0.04, Cohen’s *f* = 0.19]; long-term verbal memory [*F*_(2, 168)_ = 5.12, *p* = 0.007, *n*^2^*_p_* = 0.06, Cohen’s *f* = 0.23]; long-term verbal memory with cues [*F*_(2, 168)_ = 5.81, *p* = 0.004, *n*^2^*_p_* = 0.07, Cohen’s *f* = 0.24]; recognition [*F*_(2, 168)_ = 3.18, *p* = 0.044, *n*^2^*_p_* = 0.04, Cohen’s *f* = 0.19]; and discriminability [*F*_(2, 168)_ = 3.48, *p* = 0.033, *n*^2^*_p_* = 0.04, Cohen’s *f* = 0.20]. In these variables, vulnerable patients performed significantly worse than the low-impact group (for all, *p* ≤ 0.04), except on recognition, in which the difference did not reach statistical significance (*p* = 0.06). Furthermore, vulnerable patients had lower scores than resilient patients on long-term verbal memory and long-term verbal memory with semantic cues (*p* < 0.03).

**Figure 4:**
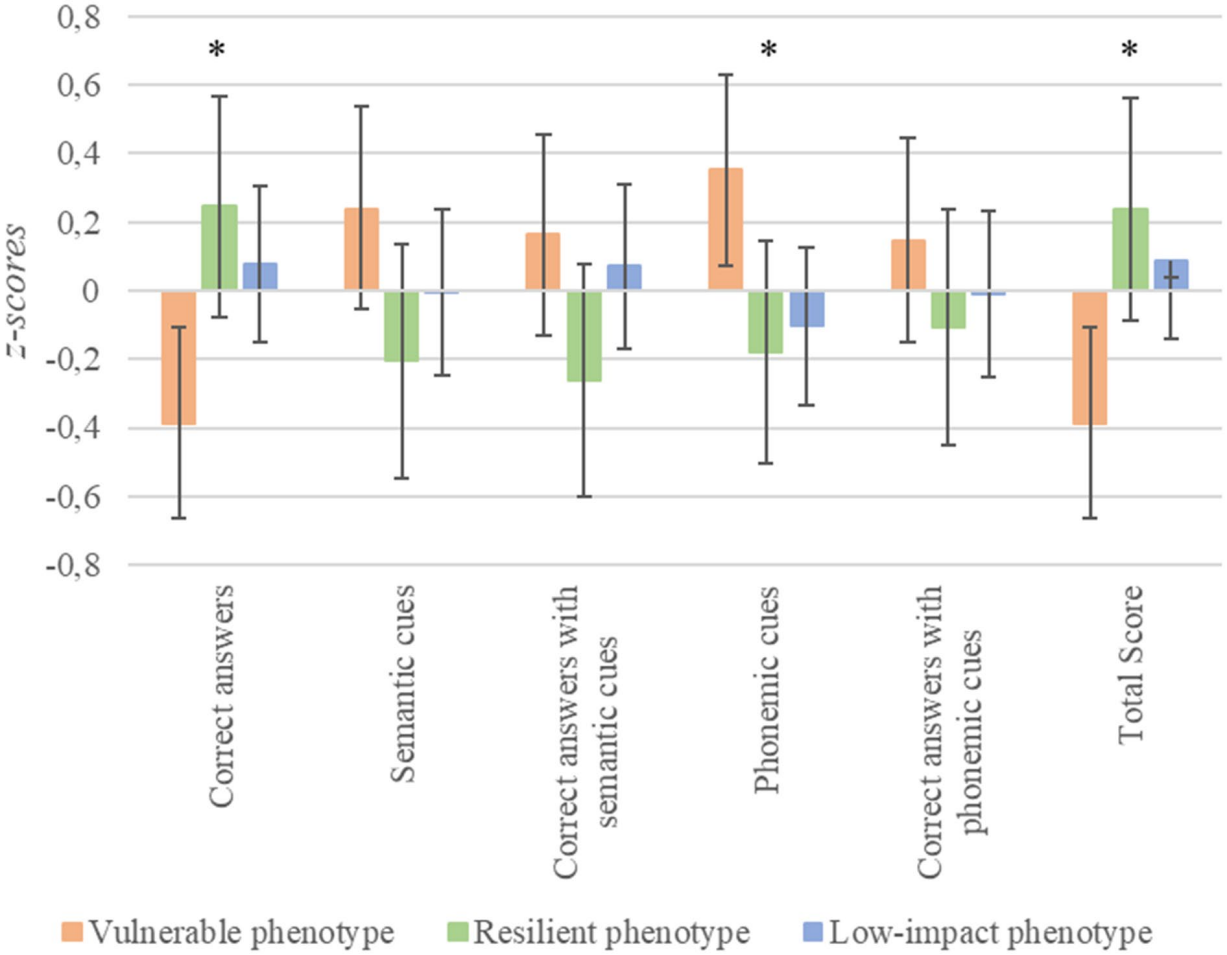
Differences in visual confrontation naming depending on stress phenotypes. ^*^p < 0.05; error bars represent 95% confidence intervals.

Finally, concerning the naming functions (Figure 4), significant differences were found in the number of correct responses [*F*_(2, 168)_ = 5.14, *p* = 0.007, *n*^2^*_p_* = 0.06, Cohen’s *f* = 0.24]; the number of phonemic cues [*F*_(2, 168)_ = 4.21, *p* = 0.017, *n*^2^*_p_* = 0.05, Cohen’s *f* = 0.22]; and the total score [*F*_(2, 168)_ = 5.20, *p* = 0.006, *n*^2^*_p_* = 0.06, Cohen’s *f* = 0.24]. In these variables, vulnerable patients performed significantly poorer than the low-impact group (for all, *p* < 0.04) and the resilient group [for all, *p* < 0.04, except for the number of phonemic cues, where a tendency was found (*p* = 0.059)].

No significant differences were found in other processes (i.e., attention, visual memory, verbal fluency).

### 3.5. Differences in QOL depending on stress phenotypes

**Figure 5:**
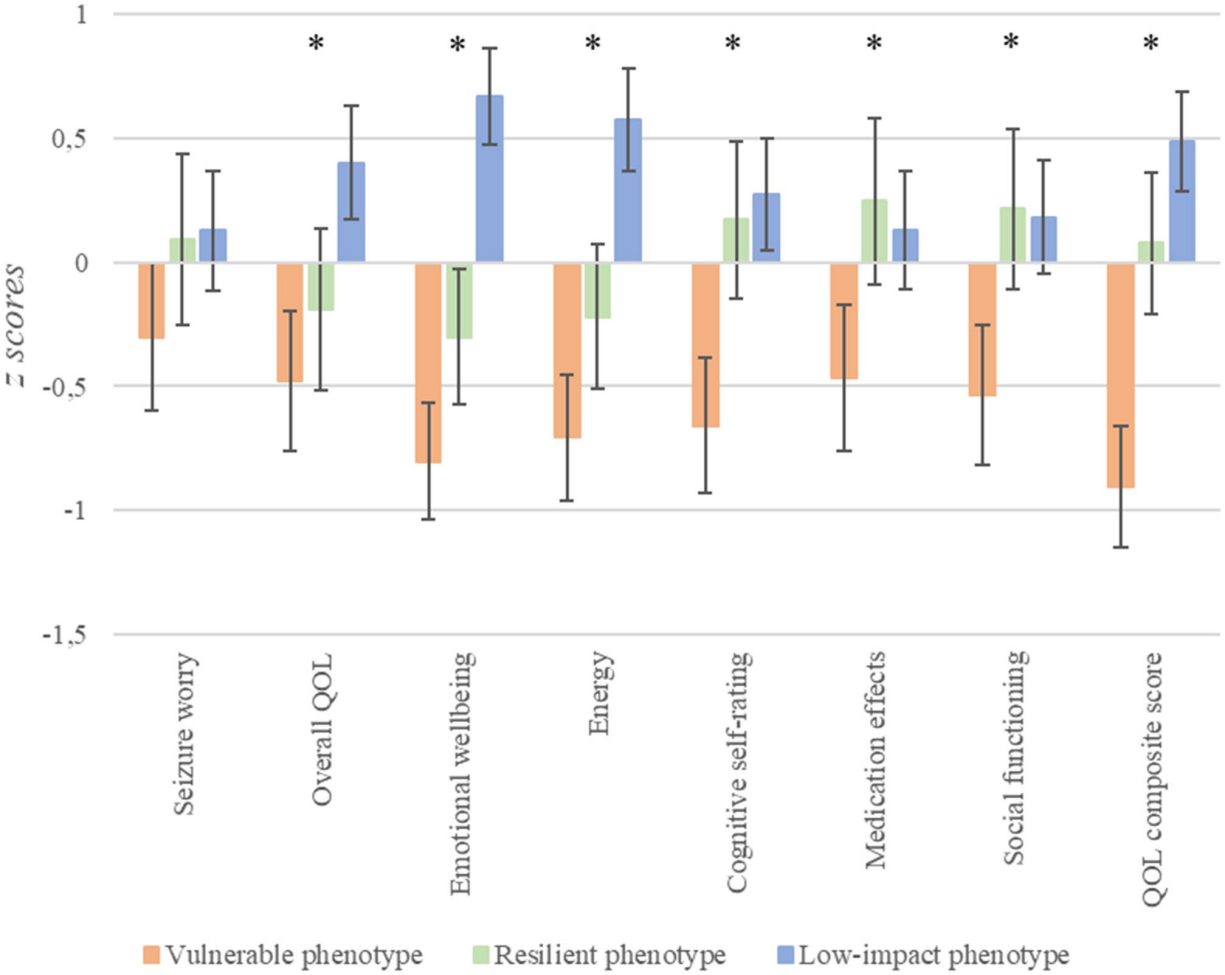
Differences in QOL depending on stress phenotypes. ^*^p < 0.05; error bars represent 95% confidence intervals.

Regarding QOL (Figure 5), significant differences were found in overall QOL [*F*_(2, 168)_ = 12.25, *p* < 0.0001, *n*^2^*_p_* = 0.13, Cohen’s *f* = 0.38]; emotional wellbeing [*F*_(2, 168)_ = 48.27, *p* < 0.0001, *n*^2^*_p_* = 0.37, Cohen’s *f* = 0.63]; energy [*F*_(2, 168)_ = 32.06, *p* < 0.0001, *n*^2^*_p_* = 0.28, Cohen’s *f* = 0.55]; cognitive self-rating [*F*_(2, 168)_ = 15.44, *p* < 0.0001, *n*^2^*_p_* = 0.16, Cohen’s *f* = 0.40]; medication effects [*F*_(2, 168)_ = 6.80, *p* = 0.001, *n*^2^*_p_* = 0.08, Cohen’s *f* = 0.29]; social functioning [*F*_(2, 168)_ = 9.60, *p* < 0.0001, *n*^2^*_p_* = 0.11, Cohen’s *f* = 0.32]; and QOL composite score [*F*_(2, 168)_ = 39.62, *p* < 0.0001, *n*^2^*_p_* = 0.33, Cohen’s *f* = 0.57]. Vulnerable patients had significantly poorer QOL than the low-impact group in all these variables (for all, *p* ≤ 0.005). Furthermore, resilient patients had significantly lower scores than the low-impact group on overall QOL (*p* = 0.024), emotional wellbeing (*p* < 0.0001), and energy (*p* < 0.0001). Finally, vulnerable patients showed poorer QOL than resilient patients on emotional wellbeing (*p* = 0.028), cognitive self-rating (*p* < 0.001), medication effects (*p* = 0.008), social functioning (*p* < 0.0001) and QOL composite score (*p* < 0.0001). It should be noted that a tendency was found for energy, with vulnerable patients having lower scores than resilient patients (*p* = 0.052). No other significant differences were found.

## 4. Discussion

The results of the current study show three stress phenotypes in patients with drug-resistant epilepsy based on indicators of stress chronicity and intensity (i.e., epilepsy duration and negative affectivity): vulnerable; resilient; and low-impact phenotypes. Our findings show that executive function, memory, naming, and QOL differ depending on stress phenotypes. These main findings are discussed below.

### 4.1. Stress phenotypes in epilepsy: characteristics

The vulnerable phenotype was characterized in our study by shorter epilepsy duration, but high levels of anxiety and depression. The resilient phenotype was characterized by long epilepsy duration and moderate anxiety and depression, suggesting that patients included in this group may have established effective coping strategies or the chronicity of the disease favored compensatory mechanisms that lead to a moderation of negative affectivity. It should be noted that this was the only group to present a long duration for the disease. A low-impact group with low anxiety, depression, and epilepsy duration was also identified. These phenotypes provide us with a new classification of patients with epilepsy in the context of epilepsy as a chronic stress setting. To our knowledge, no previous studies have established phenotypes from a quantitative research perspective considering both stress chronicity and intensity of stress in patients with epilepsy.

Our results showed that stress phenotypes were similar in terms of clinical variables such as epilepsy type, side of seizure focus, and seizure frequency. However, differences were found in MRI findings, with a higher frequency of HS in the resilient group. HS is the most common finding in epilepsy surgery (Malmgren and Thom, 2012) and so the surgery rate is high. Therefore, patients with this condition may be more likely to feel more hopeful about their futures and feel that they have more options and so, therefore, can adopt more resilient behavior. Furthermore, we found that patients in the resilient group had a lower educational level than patients in the low-impact group. The fact that patients from the resilient group had an earlier age of onset of epilepsy (i.e., longer epilepsy duration) could have hindered subsequent academic development in the resilient group with respect to the low-impact group. Furthermore, it should be noted that patients in the low-impact group had lower levels of negative affectivity than the resilient group, and some studies have found that a higher educational level is related to lower anxiety (Mensah et al., 2007; Peterson et al., 2014), so having low negative affectivity could have acted as a protective factor for educational attainment for the low-impact group.

We also found that resilient patients were significantly older and took more ASMs than the other groups. In samples of people with epilepsy, older patients were found to have healthier coping styles than younger patients (Bautista et al., 2013) and were better adapted to a chronic illness (Canuet et al., 2009). Regarding the number of ASMs, Şenadım et al. (2021) found that focusing on and venting emotions was more frequent in patients with epilepsy who were receiving polytherapy than in those receiving monotherapy (without examining the influence of the specific number of ASMs and so underlying the need for further studies to clarify this relationship). It should be noted that, in our study, most patients (95.3%) received polytherapy. The fact that resilient patients took more ASMs than vulnerable patients may be due to higher chronicity of the disease (i.e., epilepsy duration) and an earlier onset of the disease (Szaflarski et al., 2006; Witt et al., 2020). Together, these results indicate that age and number of ASMs are important factors in understanding stress phenotypes, so they were included as covariates in the further analyses together with educational level and HS.

Due to the high rate of alexithymia in patients with epilepsy (Ricciardi et al., 2015), differences between these groups in emotional recognition were studied. No significant differences were observed among the three groups, with all three presenting a hit rate of at least 50%. This suggests that the differences found in negative affect and QOL in these groups may be not due to possible difficulties in emotional perceiving.

### 4.2. Differences in attention and executive functioning depending on phenotypes

Significant differences were found in executive functioning between stress phenotypes with the resilient group performing better than the vulnerable and low-impact groups, with small-to-medium effect sizes depending on the variable considered. Although some studies have indicated that longer epilepsy duration is related to poorer performance in executive functions (Kim et al., 2007; Black et al., 2010; Zamarian et al., 2011), other studies have found that duration is a weak predictor of this variable (Thompson and Duncan, 2005; Wang et al., 2011). Some studies have found no relationship between epilepsy duration and executive functioning (Agah et al., 2017; DeGeorge et al., 2021) and our results suggest that negative affectivity, an increasing factor of stress intensity, may be more relevant to explain executive function deficits in patients with epilepsy than stress chronicity.

More detailed vulnerable patients had poorer performance in executive functions than the resilient group, despite the latter group having longer duration of epilepsy. Espinosa et al. (2010) found that WCST performance was related to the severity of depressive disorders in patients with TLE, suggesting that patients with a larger number of total errors were more likely to have depression. This finding is interesting, as significant differences in the total number of errors were only found between the resilient and vulnerable groups, without differences with the low-impact group—who had low negative affectivity despite having the same duration as the vulnerable group. Significant differences were also found in perseverative errors, which has been found to be a predictor of postsurgical BDI score in a sample of TLE patients (Pope et al., 2019). However, there are few studies that address this relationship while considering anxiety. As an exception, Hermann et al. (1991) addressed the relationship between the number of perseverative errors and negative affectivity, including measures of depression and anxiety, and found a significant association between these variables in patients with left TLE, but no significant results in patients with right TLE.

Surprisingly, we also found that the resilient group also performed better in executive function than the low-impact group—with lower perseverative errors and perseverative responses (these variables being measures of cognitive flexibility). These findings are relevant, as patients with a longer history of illness have been found to perform better than those with a shorter duration and lower emotional comorbidity. Cognitive flexibility is a domain related to problem-solving skills and decision-making, that enables the patient to develop different coping strategies to deal with a problem. Some studies that have examined cognitive flexibility with self-report questionnaires have found a positive relationship between resilience and stress coping (Martin and Anderson, 2001; Bonanno et al., 2004; Arici-Ozcan et al., 2019). Soltani et al. (2013) proposed that this relationship may be more complex, and found that cognitive flexibility mediates the relationship of coping styles and resilience with depression. These findings suggest that cognitive flexibility may interfere with the moderate levels of negative affectivity shown in this group.

It should be underlined that, although it cannot be established with certainty that the differences found in executive function tests depending on stress phenotypes were not influenced by other factors, stress phenotypes did not differ in attention functioning, so differences in executive functioning may not be attributed to deficits in this cognitive domain.

### 4.3. Differences in memory depending on phenotypes

We found significant differences in verbal memory functioning depending on stress phenotypes, with small effect sizes. Specifically, we found poorer memory functioning in the vulnerable group than in the low-impact group. This suggests that negative affectivity is more related to memory impairment than the duration of the disease itself, as both the vulnerable and low-impact groups were equal in epilepsy duration. These results are in line with previous studies that showed that trait anxiety is a predictor of memory functioning (Cano-López et al., 2019) and that patients with epilepsy with depression had cognitive impairments (Arend et al., 2018). Rayner et al. (2016a) suggested that depression may be more strongly linked to deficits in cognitive networks in patients with epilepsy than in cases of primary depression, as there may be mechanisms underlying depression that could affect brain networks in some neurological diseases.

Our results also showed significantly poorer verbal memory functioning in vulnerable patients compared to the resilient group in two subscales (i.e., long-term verbal memory and long-term verbal memory with semantic cues). It should be noted that in our sample resilient patients began to have seizures in childhood and, consequently, have longer epilepsy duration, whereas the vulnerable group began to have seizures in adulthood (with a shorter epilepsy duration). In contrast to our results, it has been found that an early onset of epilepsy may cause changes in brain development characterized by an age-related delay in white matter gain, possibly leading to problems in cognitive development (Hermann et al., 2010), and that epilepsy duration and age of epilepsy onset are predictors of memory failure in children (Menlove and Reilly, 2015) and adults with TLE (Uslu et al., 2019). However, it should be noted that some studies have found memory deficits in patients with a recent diagnosis of epilepsy not treated with ASMs, suggesting that memory impairment in patients with long-standing epilepsy cannot be attributed exclusively to seizure recurrence and the effects of ASMs, and other factors must be explored (Aikia et al., 2001). It is also possible that the early onset of the resilient group could favor a better performance in the adult stage due to the efficiency of the plasticity and compensatory mechanisms in childhood and adolescence. In our study, vulnerable patients had more negative affectivity than resilient patients, so our results suggest that it is not so much the duration of epilepsy that matters, but how it combines with negative affectivity to explain the differences between the resilient and vulnerable phenotypes in memory functioning.

Rayner et al. (2016b) show that memory failures could be explained depending on the age at epilepsy onset. In this regard, memory failures in patients with early epilepsy onset are mainly explained by neurobiological factors (i.e., the resilient group in our sample), whereas in patients with late-onset these deficits are due to psychological maladaptation (i.e., vulnerable and low-impact groups). Our findings partially agree with this model, as we conclude that in patients with late epilepsy onset, memory failures are mainly related to negative affectivity. In our study, the group with high negative affect and short epilepsy duration (vulnerable group) performed worse than the group with low negative affect and low epilepsy duration (low-impact group) on all memory subscales. Furthermore, these differences were also significant for the long-term memory subscales between the group with moderate levels of negative affect and long epilepsy duration (resilient group) and the vulnerable group (with the vulnerable group showing poorer performance). These results suggest that negative affect is a sensitive factor that may have a stronger effect on memory than epilepsy duration.

It should be noted that no significant differences were found in visual memory depending on stress phenotypes. This could be due to the type of task used to evaluate visual memory, as there are difficulties in finding a ‘pure’ test of visual memory (Helmstaedter et al., 1995; Vaz, 2004), and patients often use verbal strategies to perform visual tests such as the ROCF (Barr et al., 1997).

### 4.4. Differences in language functioning depending on phenotypes

Significant differences were found in visual confrontation naming, with small effect sizes. Specifically, the vulnerable group showed poorer performance than the low-impact group and the resilient group. Vulnerable and low-impact groups had similar epilepsy duration, so our results highlight the relevance of negative affectivity in naming and semantic memory processes. Furthermore, the fact that the vulnerable group (i.e., high negative affectivity and short epilepsy duration) had also poorer performance than the resilient group (i.e., moderate negative affectivity and long epilepsy duration) in naming suggests that naming deficits in patients with late epilepsy onset are mainly associated with negative affectivity. These results are in line with those found by Paradiso et al. (2001), showing that patients with TLE and depression had poorer performance than patients with TLE without depression in a neuropsychological evaluation, in which visual naming was assessed. As indicated above, mechanisms underlying negative affectivity may affect brain networks involved in cognitive functioning (Rayner et al., 2016a).

Decreased naming ability is commonly found in patients with epilepsy, especially in those with TLE, indicating a key role of the medial temporal lobe in this process (Goodglass and Wingfield, 1997; Ogden-Epker and Cullum, 2001; Schefft et al., 2003; Bonelli et al., 2011). It should be noted that stress phenotypes did not differ in verbal fluency, suggesting that the differences found in naming may be attributed to semantic memory functioning and not to other processes such as verbal fluency. Taken together, our results are in line with those found with episodic verbal memory, pointing to an interrelation between semantic and episodic memory (Cano-López et al., 2017; Barrett Jones et al., 2022).

### 4.5. Differences in QOL depending on phenotypes

In terms of QOL, significant differences with medium-to-large effect sizes (depending on the QOL score considered) were found depending on stress phenotypes. Specifically, we found that the vulnerable group had poorer QOL than the low-impact group in QOL composite score and most QOL subscales. These findings are in line with several studies that showed that depressive symptoms and anxiety are negatively related to QOL (Kwan et al., 2009; Gur-Ozmen et al., 2017; Micoulaud-Franchi et al., 2017; Scévola et al., 2017; Cano-López et al., 2018; Tombini et al., 2020b; Lima et al., 2021), with anxiety even being the strongest predictor of QOL in patients with TLE (Cano-López et al., 2018; Lima et al., 2021).

Our results also showed that the resilient group had poorer QOL than the low-impact group in overall QOL, emotional wellbeing, and energy subscales. Regarding overall QOL, some studies have found that epilepsy duration is related to poorer scores in this subscale in people with epilepsy (Shetty et al., 2011; Pauli et al., 2012). We argue that patients with longer durations may have a more comprehensive affectation of the disease that may affect different areas, and therefore they may have poorer overall QOL. Regarding emotional wellbeing, it should be noted that the low-impact group was taken as a representation of patients who did not yet have emotional alterations associated with epilepsy. However, the resilient group was not completely exempt from these emotional alterations (i.e., having moderate levels of negative affectivity). This may explain why patients from the resilient group, with moderate levels of anxiety and depression, have less QOL related to emotional wellbeing than the low-impact group. Regarding the energy subscale, our results may be influenced by the longer epilepsy duration that characterized resilient patients compared to patients from the low-impact group, considering that recurrent exposition to seizures may worsen the signs of fatigue associated with age and chronic illness in patients with epilepsy (Baranowski, 2018).

We also found a significantly poorer QOL in the vulnerable group compared to the resilient group in all the QOL subscales, except for seizure worry and overall QOL (where no differences were found) and energy (where a tendency was found). Regarding emotional wellbeing, since the vulnerable group had a higher negative affectivity than the resilient group, it was to be expected that they would perceive a lower related QOL. As regards cognitive self-rating, the vulnerable group had poorer objective memory scores than the resilient group, but the differences did not reach statistical significance for most variables. This suggests that differences between vulnerable and resilient groups are more notable in subjective cognitive performance than in objective cognitive functioning, which is congruent with the high negative affectivity scores of the vulnerable group. In the case of medication effects, we found that the resilient group, despite taking the largest number of ASMs, perceived fewer medication effects than the vulnerable group. This contrasts with studies that showed that the number of ASMs is related to QOL (Lozano-Garcia et al., 2021) even when depression was considered in this relation (Wang et al., 2022). However, other studies have found that patients with comorbid depression (Josephson et al., 2017) or anxiety (Kanner et al., 2012) experience greater adverse effects from ASMs. This could explain, at least in part, why patients with higher negative affectivity experience worse adverse effects and, consequently, a poorer related QOL. Regarding social functioning, the vulnerable group had poorer scores than the resilient group. This may be explained by two facts. Firstly, vulnerable patients with a shorter period of adaptation to the disease may feel more stigmatized and may perceive less seizure control and a greater sense of unpredictability, which may be associated with greater social isolation and, therefore, with reduced QOL (Suurmeijer et al., 2001; Błaszczyk and Czuczwar, 2016). Secondly, the vulnerable group had higher negative affectivity, and this could be related to social problems. In this line, a study of patients with TLE found that anxiety was negatively related to both perceived social support and social functioning (Catalán-Aguilar et al., 2022). Furthermore, it has been shown that social support plays an important role as a predictor of negative affectivity in epilepsy (Gandy et al., 2012).

### 4.6. Strengths and limitations of this study

This study has strengths such as the large and relatively homogeneous sample (i.e., adult patients with drug-resistant epilepsy). Although Ring et al. (2016) differentiated vulnerable and resilient individuals and analyzed how different socioemotional and clinical factors influence QOL in patients with epilepsy using qualitative methods, our study represents an advance over previous studies, since, as far as we know, it is the first to summarize stress chronicity and intensity variables in different profiles from a quantitative research perspective and to explore their association with cognition and QOL in patients with epilepsy. Our findings highlight that it is not so much the epilepsy duration and negative affectivity separately that are relevant, but how these variables combine to form phenotypes. It is worth noting that the anxiety assessed in this study is the anxiety trait and not a scale of clinical anxiety. This enhances the predictive power of this dimension in the detection of vulnerable and resilient individuals since it is not necessary to reach clinical scores. Hermann et al. (2021) recommended that future studies move toward a new taxonomy of epilepsy, in which cognitive and psychological comorbidities become more relevant. Considering this suggestion, we proposed new phenotypes according to emotional comorbidity and epilepsy duration. In addition, to ensure that other comorbidities such as memory deficits, executive functioning impairment, naming difficulties or poor QOL are examined, we show differences depending on stress phenotypes in these variables.

Despite these strengths, some limitations should be considered. First, although our sample was composed of patients with drug-resistant epilepsy sample, different types of epilepsy were included. For this reason, future studies should explore the identified phenotypes with specific types of epilepsy. Second, due to the cross-sectional nature of the data, we cannot conclude causality in the relationships. Third, larger sample sizes could provide more information and ensure statistical power. Fourth, executive function is a complex cognitive domain, so the inference of executive function only from WCST and TMT scores should be considered with caution (Miranda et al., 2020). Fifth, although negative affectivity has been considered in stress phenotypes and may summarize the heightened emotional response to unpredictable seizures, the unpredictability of seizures was not registered, so further studies may design new measurements for this variable. In this line, stress indicators such as cortisol levels or response are also needed to confirm the stress in these three phenotypes and future research in this line must be encouraged. Sixth, although side of seizure focus is a variable of great interest for understanding the idiosyncrasies of epilepsy, it was not considered in determining stress phenotypes since its relationship with stress processes is inconsistent. Specifically, some studies showed that patients with left TLE have higher levels of depression and anxiety (Altshuler et al., 1990), whereas others found a lack of relationships of side of seizure focus with negative affectivity (Devinsky et al., 2005; Sperli et al., 2009; Cano-López et al., 2019) or cortisol response (Cano-López et al., 2019). This highlights the need for further studies to assess the possible relationship between stress and lateralization of epilepsy. Seventh, the group labels of “resilient,” “vulnerable,” and “low-impact” must be taken cautiously since they are supported by criteria of negative affectivity and duration of epilepsy. This does not mean that resilient patients show better outcomes or vulnerable patients display worse outcomes in all the assessed dimensions. On the contrary, these phenotypes point to the strengths and weaknesses of each type of patient to better outline the necessary interventions. Eighth, although our anxiety and depression scores could be useful for detecting susceptibilities to clinical disorders (Cano-López et al., 2019) they are not diagnostic measures (Kendall et al., 1987). We used the BDI and STAI to evaluate negative affectivity since data collection began in 2015 using these tests and it was decided to continue using the same instruments for reasons of comparability, but future studies should replicate these results with screening tools specifically designed and validated in patients with epilepsy for depressive disorders and anxiety disorders. Finally, to ensure the ability of the participants to recognize one’s own emotions, future studies should consider including a measure of alexithymia that allows for the assessment of the possible difficulties in emotional self-recognition.

### 4.7. Conclusion

The results of the current study contribute to differentiating phenotypes based on stress chronicity and intensity, while considering both epilepsy duration and negative affectivity. Vulnerable patients have poorer memory, naming, and QOL. This group of patients is the most clinically relevant, as it corresponds to a risk profile. This indicates the need to detect non-clinical anxiety and depression in the early years of epilepsy. Patients in the resilient group perform better on executive functions. These patients have experienced epilepsy practically all their lives, adapting the disease to their environment. However, this does not imply better memory functioning and QOL than the low-impact group. Finally, it should be noted that participants from the low-impact group had a low negative affect and a similar level of memory to those from the resilient group, and had a higher QOL than the rest of the groups. These findings show the importance of taking into account negative affectivity and memory functioning during the early years of the disease, whereas in later stages executive functions become more relevant. Furthermore, these results highlight the relevance of considering epilepsy as a model of interconnected networks, in which comorbidities and clinical and demographic variables interact with each other. In fact, people with epilepsy perceive poor life satisfaction, probably due to the interaction between low perceived QOL, socio-emotional limitations, daily challenges, and the effects of the disease itself (Kang, 2023). Resilience is not a static variable, but a dynamic progress (Luthar and Cicchetti, 2000; Hatala et al., 2013), so our results could be useful for detecting vulnerable or resilient profiles and implementing individualized clinical treatments that are more tailored to patient needs. Wagner et al. (2010) demonstrated the benefits of promoting resilience in patients with epilepsy. Future research with patients with epilepsy should examine stress chronicity and intensity variables together with specific coping instruments, as well as study the relevance of possible modulating factors such as social support. Finally, we encourage assessing the efficacy of interventions designed to enhance resilience in patients with drug-resistant epilepsy.

## Data availability statement

The raw data supporting the conclusions of this article will be made available by the authors, without undue reservation.

## Ethics statement

The studies involving human participants were reviewed and approved by Ethics Committee of the Hospital Universitario y Politécnico La Fe, Valencia, Spain. The patients/participants provided their written informed consent to participate in this study.

## Author contributions

VV and KH established the diagnosis of drug-resistant epilepsy, recruited the patients for neuropsychological evaluation, and revised the manuscript. JC-A, AL-G, and PT-P participated in the neuropsychological assessment and interpretation of the data. JC-A managed the literature search. JC-A and KH undertook the statistical analyses. JC-A, EG-B, and IC-L interpreted the results, revised the literature, and wrote the manuscript. All authors contributed to the article and approved the submitted version.

## Funding

This work was supported by the project PID2020-118992RB-I00 funded by MCIN/AEI/10.13039/501100011033. JC-A was supported by the Generalitat Valenciana (Valencian Government) (grant no. ACIF/2021/094).

## Conflict of interest

The authors declare that the research was conducted in the absence of any commercial or financial relationships that could be construed as a potential conflict of interest.

## Publisher’s note

All claims expressed in this article are solely those of the authors and do not necessarily represent those of their affiliated organizations, or those of the publisher, the editors and the reviewers. Any product that may be evaluated in this article, or claim that may be made by its manufacturer, is not guaranteed or endorsed by the publisher.

## Footnotes

^1^ https://www.r-project.org/
